# Tensile Behavior Assessment of Grid-Type CFRP Textile-Reinforced Mortar with Different Design Variables

**DOI:** 10.3390/ma17246049

**Published:** 2024-12-10

**Authors:** Jung-Il Suh, Sung-Woo Park, Kyung-Min Kim

**Affiliations:** Construction Technology Research Center, Construction Division, Korea Conformity Laboratories, 199, Gasan Digital 1-ro, Geumcheon-gu, Seoul 08503, Republic of Korea; rgtonesuh@kcl.re.kr (J.-I.S.); sungwoo@kcl.re.kr (S.-W.P.)

**Keywords:** carbon-fiber-reinforced polymer, textile-reinforced mortar, grid-type fiber-reinforced polymer reinforcement, tensile behavior assessment, surface treatment

## Abstract

This study investigates the tensile behavior of carbon-fiber-reinforced polymer (CFRP) and textile-reinforced mortar (TRM) under various design variables to enhance understanding and application in construction structures. TRM reinforced with CFRP grids is highly effective for strengthening existing structures due to its lightweight nature, durability, ease of installation, and corrosion resistance. The research aims to evaluate how design parameters such as the CFRP grid type, mortar matrix strength (influenced by the water-to-cement ratio), specimen length, and grid width affect TRM’s mechanical properties. Through the direct tensile test using a universal testing machine, TRM specimens were subjected to load until failure, with data collected on stress–strain relationships, crack patterns, and strengths. Specimens included untreated CFRP grids (Groups KC, Q47, and Q85) and sand-coated CFRP grids (Specimens AQ47_7 and AQ85_7), each tested under controlled laboratory conditions. The results indicate that crack formation significantly influenced load transfer mechanisms within the specimens, with longitudinal strands bearing load as cracks propagated through the mortar matrix. The presence of sand-coated CFRP grids notably enhanced interfacial bond strength, leading to increased cracking strength and ultimate strength compared with their untreated counterparts. The findings underscore the importance of the surface treatment of CFRP grids for improving TRM performance, with implications for enhancing structural integrity and durability in practical applications. The results provide valuable insights into optimizing TRM design for better crack control and mechanical efficiency in infrastructure.

## 1. Introduction

Reinforced concrete (RC) is the most commonly used composite material in structural construction. However, exposure to various environmental conditions can lead to durability issues due to corrosion when rebar is exposed to moisture and air [1,2]. To address the durability reduction in reinforced concrete structures caused by rebar corrosion, various attempts have been made to replace rebar [3,4]. For example, studies [5,6,7,8] investigated the use of fiber-reinforced polymer (FRP) as tensile reinforcement for concrete. According to the American Concrete Institute (ACI) 224 requirements [9], the crack width required under service load for various exposure conditions should range from 0.1 to 0.4 mm, demonstrating the efficiency of high-strength textile materials [10,11].

Textile-reinforced mortar (TRM) is a new type of cement composite consisting of a continuous grid-like textile and a mortar matrix. The application of resin-impregnated textile, i.e., FRP, in TRM is considered a promising and effective solution for strengthening existing RC and masonry structures due to its high strength-to-weight ratio, easy installation, and excellent corrosion resistance [12,13,14]. Researchers involved in the development and application of new materials have systematically investigated the mechanical properties of TRM [15,16,17,18]. Furthermore, they have evaluated its field applicability through the application of new structural members utilizing TRM [19,20,21].

Some studies related to TRM have focused on grid-type carbon-fiber-reinforced polymer (CFRP) reinforcements due to their availability and performance [22]. The integration between grid-type CFRP reinforcement and the mortar matrix is crucial for the tensile properties of TRM. The tensile behavior of TRM is determined by the interfacial compatibility, specifically the surface bond between the grid-type CFRP reinforcement and the mortar matrix. Common textile reinforcements exhibit poor interfacial compatibility with cementitious materials [23]. This often leads to slippage of textile reinforcement and delamination of the mortar matrix, which are common failure modes that cause inconsistent data dispersion [5,24].

To enhance the bond strength between the FRP and the mortar matrix, various physical treatments have been explored. Representative treatments include adding dispersed fibers, such as polyethylene [25], micro-amorphous [26], or polyvinyl alcohol fibers [27], to the mortar matrix. These fibers facilitate crack bridging. Additionally, coating the FRP surface with silica or sand improves the bond strength with the mortar matrix, resulting in improved TRM strength and more consistent performance [14,28,29].

Various testing methods for evaluating the tensile properties of TRM have been proposed. AC434 [30] focuses on textile reinforcement not impregnated with resin, while RILEM TC 232-TDT [31] and ACI 549.4R [32] target textile reinforcement impregnated with resin. These testing methods propose different lengths, widths, and thicknesses for specimens, and the tensile test processes also differ slightly. Specifically, a minimum number of strands within the specimen cross section is sometimes suggested [30]. Recently developed grid-type CFRP reinforcement varies in its manufacturing methods, the spacing of longitudinal and transverse strands, and the components of the strand cross section according to the application target and range [15,29]. Therefore, verifying whether the evaluation of the tensile behavior of TRM—reflecting the detailed specifications of the grid-type CFRP reinforcement—is suitable for the proposed testing methods is crucial.

This study investigated the impact of various design variables for the tensile testing of TRM specimens on tensile properties. Grid-type CFRP was used as the textile reinforcement, and the design variables included the types of CFRP grid (adhesive structure and biaxial warp knitting structure), strength of the mortar matrix, measuring length of TRM specimens, and strand spacing of the CFRP grid. Based on these design variables, the direct tensile test of TRM was performed following RILEM TC 232-TDT [31] to evaluate mechanical performance and define the final failure mode based on the final damage results. The bond characteristics between the CFRP grid and the mortar matrix were evaluated by summarizing the crack characteristics and relating them to the mechanical properties.

## 2. Materials and Methods

### 2.1. Materials and Specimens

**Figure 1:**
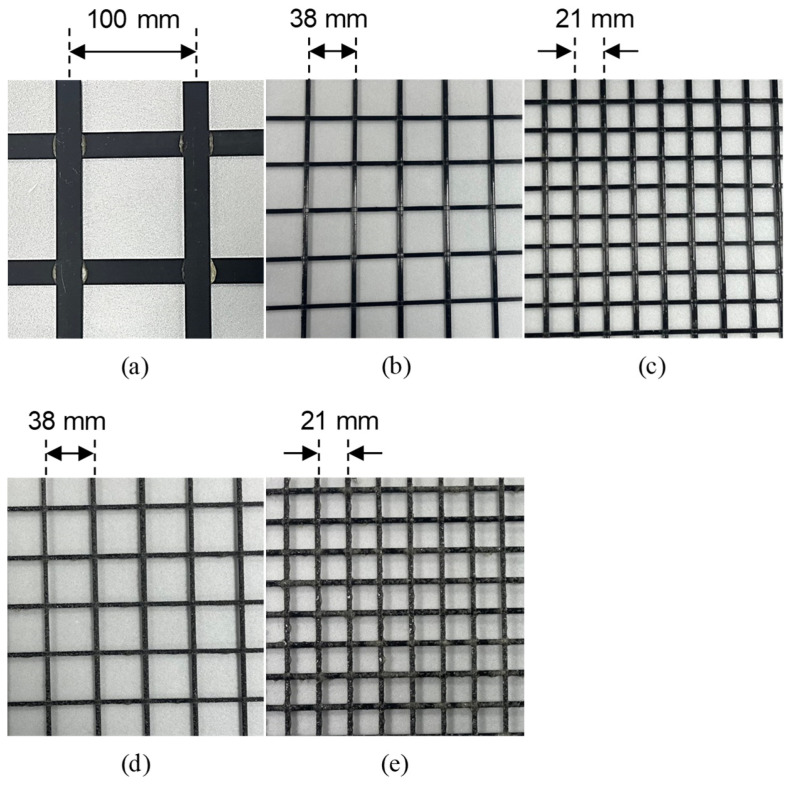
Shape of CFRP grid: (**a**) KC, (**b**) Q47, (**c**) Q85, (**d**) AQ47, and (**e**) AQ85.

**Figure 2:**
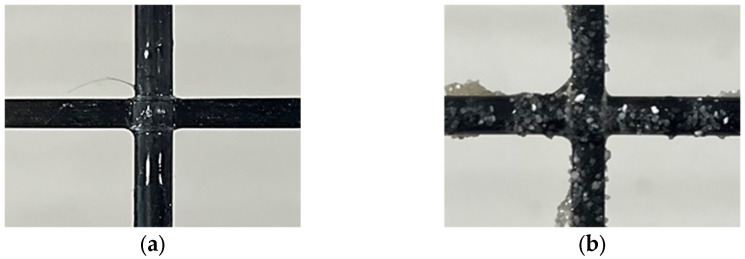
Surface treatment of CFRP grid: (**a**) untreated surface and (**b**) sand-coated surface.

The primary materials used to produce the TRM with CFRP grid as tensile reinforcement in this study were CFRP grids with adhesive structures [30] and biaxial warp knitting structures [29]. The characteristics of the CFRP grids used are listed in Table 1. The CFRP grid (KC) was manufactured by arranging thin flat strands of CFRP—20 mm wide and 1 mm thick—made from carbon fiber and epoxy in a pultrusion process in the weft and warp directions at 100 mm intervals. The intersections of the weft and warp strands were bonded with a high-strength, quick-setting adhesive of 20 MPa [33]. To compare with CFRP grid KC, four commercially available products with biaxial warp knitting structures—Q47, Q85, AQ47, and AQ85 (Solidian GmbH, Albstad, Germany)—were used (Figure 1). Specifically, as shown in Figure 2, the surfaces of AQ47 and AQ85 are coated with sand to enhance bonding behavior with cementitious materials. KC demonstrated a tensile strength of 2327 MPa and an elastic modulus of 179 GPa, whereas Q47 and AQ47 exhibited higher tensile strengths of 4389 MPa and elastic moduli of 255 GPa. Similarly, Q85 and AQ85 showed tensile strengths of 4121 MPa and elastic moduli of 244 GPa.

Based on the recommendation of RILEM TC 232-TDT [31], which focuses on textile reinforcement impregnated with resin, TRM tensile specimens for Groups KC, Q47, Q85, AQ47, and AQ85 were designed and fabricated in a slender prismatic shape with rectangular cross sections. The detailed design elements are summarized in Table 2. The group and specimen names were designated to reflect the cross-sectional area of the CFRP grid, sand coating for anti-cracking, length of TRM specimens, and water-to-cement (w/c) ratio of the mortar matrix. For instance, in “AQ47_5_0.3”, “A” denotes an application of sand coating on the grid surface for anti-cracking, “Q47” indicates a fiber cross-sectional area of 47 mm^2^ per meter, “5” signifies a specimen length of 500 mm, and “0.3” indicates a w/c ratio of 0.3.

**Figure 3:**
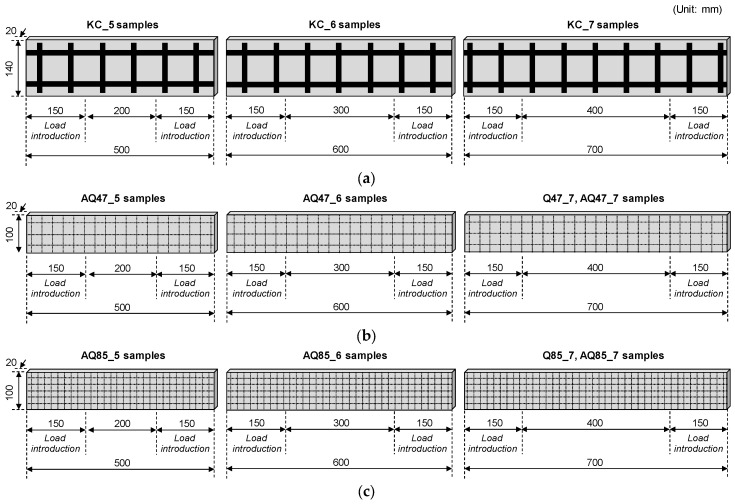
Details of TRM specimens: (**a**) Group KC, (**b**) Groups Q47 and AQ47, and (**c**) Groups Q85 and AQ85.

The primary design variables of the TRM specimens include the type of CFRP grid, the strength of the mortar matrix, the measuring length of TRM specimens, and the strand spacing of the CFRP grid (Table 2 and Figure 3). Following the direct tensile test method outlined in RILEM TC 232-TDT [31], the specimens designed for this study were a minimum of 500 mm in length and at least 100 mm wide, with a length-to-width ratio of at least 5:1. To ensure strain-hardening behavior, each specimen included at least two longitudinal strands in the cross section.

For Group KC, with a strand spacing of 100 mm and including two longitudinal strands, the specimen width was designed to be 140 mm. The specimen widths for Groups Q47 and AQ47, as well as Q85 and AQ85—incorporating three and five longitudinal strands, respectively—were designed to be 100 mm. The thickness of all TRM specimens was uniformly set at 20 mm.

The specimen lengths were 500, 600, and 700 mm in total. However, for Groups Q47 and Q85, only a length of 700 mm was considered, following the recommendation of RILEM TC 232-TDT [31]. The load introduction area, where tensile load was applied, was 150 mm at both ends, and the measuring net lengths, where the tensile behavior of the specimens is measured, were 200, 300, and 400 mm.

**Figure 4:**
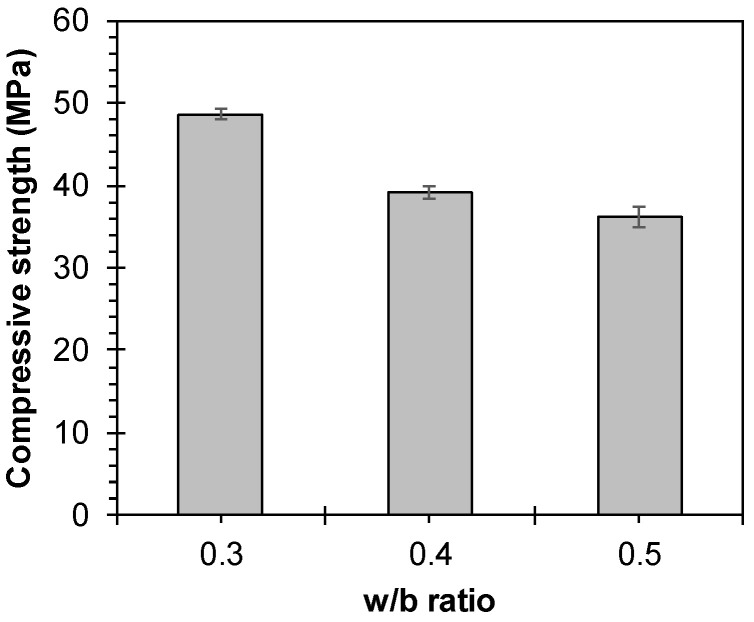
The 28-day compressive strength of the mortar.

Considering the thickness and strand spacing of the specimens, mortar served as the primary filler for TRM. The cement-to-sand mass ratio in the mortar mixture was 1:3, and the w/c ratio was determined as 0.3, 0.4, and 0.5 based on experimental variables. The materials, prepared according to ASTM C305 [34], were mixed and the mortar blend cast into 40 mm × 40 mm × 160 mm prismatic molds for measuring compressive strength. After one day of casting, the specimens were demolded and cured under laboratory conditions over 28 days before the compressive tests. The results of the 28-day compressive strength measurements (Figure 4) indicated that the compressive strengths were 48.6, 39.1, and 36.2 MPa for w/c ratios of 0.3, 0.4, and 0.5, respectively, showing a decrease with an increasing w/c ratio.

### 2.2. Test Methods

**Figure 5:**
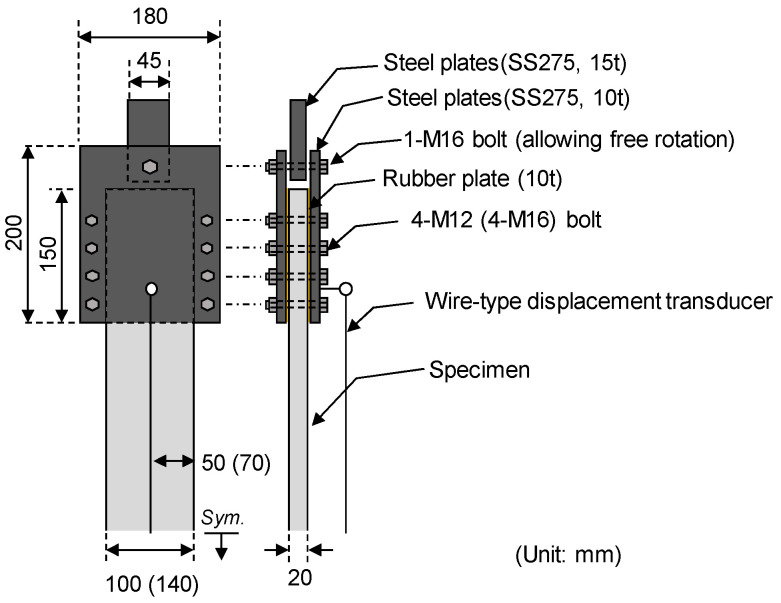
Tensile test setup of TRM specimens. The numbers in parentheses indicate the details of Group KC.

Figure 5 illustrates the direct tensile test setup for TRM specimens. The tensile behavior of these specimens was evaluated using a 1000 kN capacity universal testing machine (UTM) following the direct tensile test method recommended by RILEM TC 232-TDT [31]. Given the potential variations in strength, even among specimens with identical variables, empirical data were collected from triplicate specimens for each variable.

Steel plates (SS 275, 15t, and 10t) secured with bolts (M16 and M12) clamped the load introduction areas at both ends of the TRM specimens. The maximum bearing capacity is 457 kN for the 8-M12 bolts and 609 kN for the 8-M16 bolts. To prevent slippage in these areas, a 10 mm rubber plate (urethane) was interposed between the steel plate and the specimen.

The clamping steel plates affixed to both ends of the TRM specimens were connected to the UTM for rotational alignment, and loading occurred at a rate of 5 mm/min. A load cell integrated into the UTM measured the applied load on the TRM specimens, while changes in length within the measuring area were recorded using a wire-type displacement transducer (WTDT) affixed to the center of the steel plate.

## 3. Results and Discussion

### 3.1. Crack Pattern and Failure Mode

**Figure 6:**
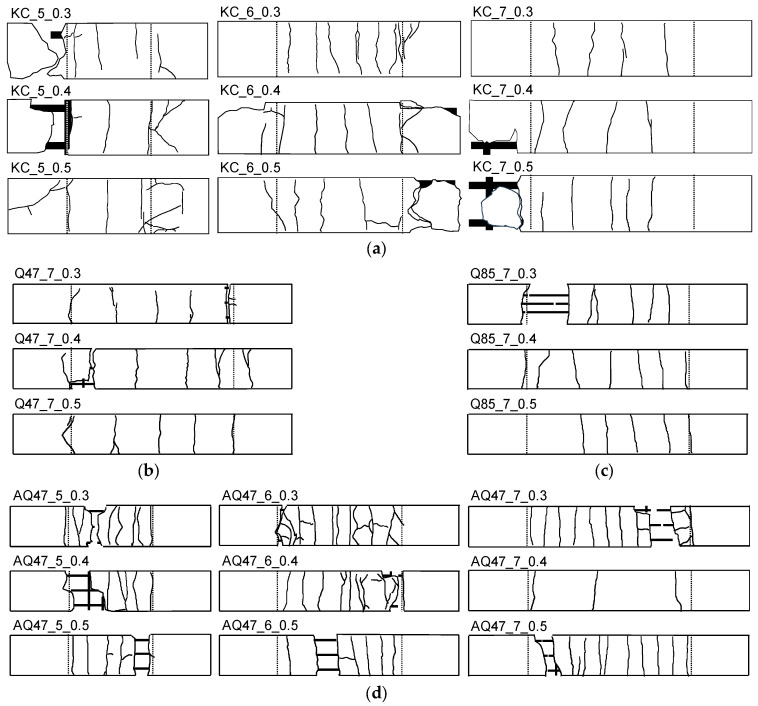

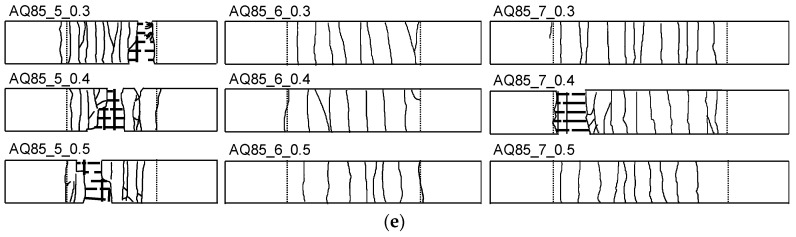
Final crack patterns of representative TRM specimens in Group (**a**) KC, (**b**) Q47, (**c**) Q85, (**d**) AQ47, and (**e**) AQ85.

Figure 6 illustrates the crack patterns observed in representative TRM specimens categorized by the type of CFRP grid following testing. In operational TRM structures, the crack spacing and the stress distribution on the textile reinforcement significantly influence the crack pattern, directly impacting structural durability.

For Groups KC, Q47, and Q85 (Figure 6a–c, respectively), the specimens exhibited fractures primarily in the central or end sections, characterized by a limited number of cracks with wide spacing. The manufacturing method of the CFRP grid, strand cross-sectional area, and strand spacing did not markedly affect the crack pattern. In Group KC, despite a high strand cross-sectional area, the 100 mm strand spacing confines crack control to a smaller area. Conversely, Groups Q47 and Q85, featuring lower strand cross-sectional areas but narrower strand spacings of 38 mm and 21 mm, respectively, demonstrated enhanced crack control areas.

Unlike the specimens from other groups, Group KC frequently exhibited longitudinal strand rupture and mortar delamination, particularly in the load introduction area. This was attributed to lower bond strength between the longitudinal strands and the mortar matrix compared with other groups, resulting in crack formation and mortar delamination due to the concentration of compressive forces from the clamping steel plate fastening and tensile loads in the load introduction area.

From the crack pattern observations of Groups AQ47 and AQ85, the sand coating on the surface of CFRP grids significantly influenced the crack pattern (Figure 6d,e). Despite having the same number of longitudinal strands, sand-coated CFRP grids showed increased crack occurrence and reduced crack spacing. The pattern of tensile cracks closely mirrored the positioning of the transverse strands. Following the final tensile test, numerous instances of mortar delamination from the CFRP grid surface were observed within the measuring area for tensile behavior. This was attributed to reduced bond strength between the longitudinal strands and the mortar matrix during high-stress stages [14,28], resulting in bond cracks, interface separation, and eventual mortar delamination.

**Figure 7:**
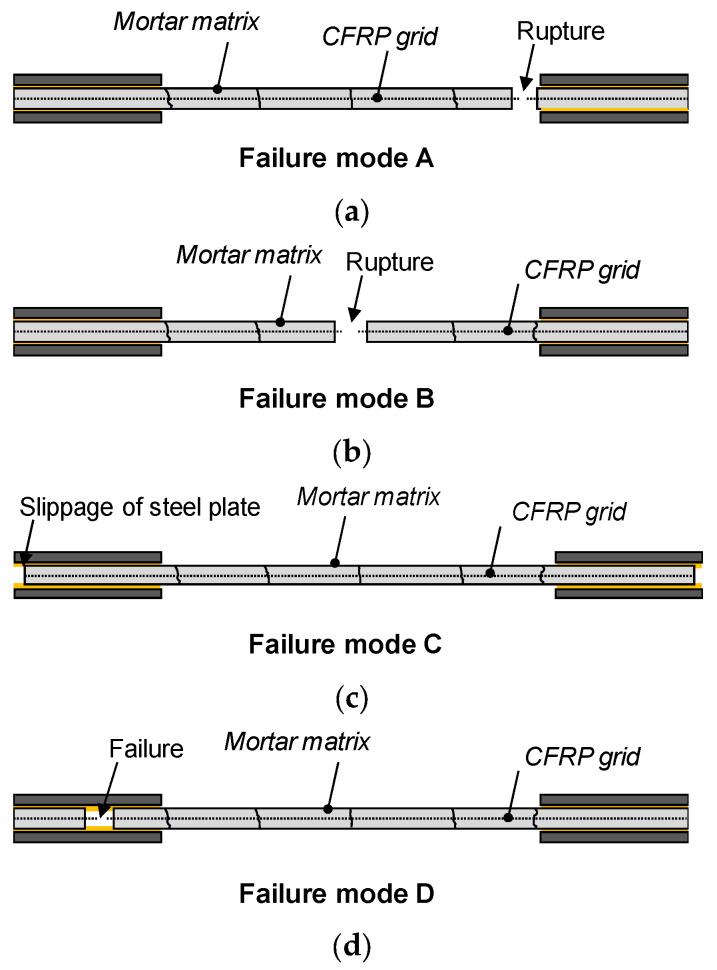
Idealized failure modes of TRM specimens: (**a**) longitudinal strand rupture near the load introduction area, (**b**) longitudinal strand rupture at the center, (**c**) slippage between clamping steel plates and specimen, and (**d**) failure at the load introduction area [15,17,35,36].

Table 3 shows the final crack patterns of all TRM specimens, determining the failure mode by referring to the idealized failure modes in Figure 7 and listing the number of cracks and average crack spacing for each specimen.

Figure 7 illustrates four distinct failure modes, A, B, C, and D, observed during the tensile tests of TRM specimens from previous studies [15,17,35,36], including a newly identified mode, D. Failure mode A (Figure 7a) depicts the final longitudinal strand rupture occurring near the specimen end adjacent to the load introduction area. This is attributed to biaxial stress at the end caused by a combination of compressive force from the clamping and applied tensile force. Failure mode B involves longitudinal strand rupture at the central part of the specimen (Figure 7b) occurring after the cracking of the mortar matrix along the specimen’s length and fiber rupture. Failure mode C (Figure 7c) shows a final failure pattern resulting from slippage of the specimen within the clamping area of the load introduction section. Failure mode D (Figure 7d) presents a newly identified pattern where the final fracture is caused by cracks and delamination of the mortar matrix within the load introduction area, which is clamped by steel plates at the specimen end. This failure results from the combined compressive and tensile load concentration from bolt fastening, along with the tensile load acting on the specimen, leading to cracks and delamination within the mortar matrix. In failure modes A and D, the interaction of compressive and tensile loads at the ends of the TRM specimen within the load introduction area is similar. The distinction lies in whether the failure occurs due to longitudinal strand rupture or mortar failure inside the load introduction area.

Most specimens exhibited failure modes A and B, although those lacking sand coating on the surface occasionally displayed failure mode C due to slippage between the clamping steel plate and TRM specimens. Specifically, Group KC frequently showed longitudinal strand rupture and mortar delamination in the load introduction area, resulting in failure mode D, unlike the specimens from other groups. This was attributed to lower bond strength between the longitudinal strand and mortar compared with other groups.

The number of cracks was visually observed within the measuring range, and the average crack spacing (*s_m_*) was calculated according to Equation (1) [37]:

(1) $$s_{m}=\frac{l_{\mathrm{TRM}}}{n_{\mathrm{crack}}-1}$$

where *l_TRM_* is the measuring net length of the specimen and *n_crack_* is the number of cracks formed in the measuring range along the specimen.

After the tensile test, Group KC exhibited 3–7 cracks in the measuring range, with an average crack spacing of 50.00–100.00 mm. Considering the strand spacing of Group KC is 100 mm, many cracks occurred below the transverse strand spacing distance.

Comparing Groups Q47 and Q85 with the 700 mm long specimens AQ47_7 and AQ85_7, Groups Q47 and Q85 exhibited 4–7 cracks within the measuring range, resulting in an average crack spacing of 66.7–133.3 mm. Conversely, the sand-coated specimens AQ47_7 and AQ85_7 showed more than twice the number of cracks compared with the Groups Q47 and Q85, thereby the average crack spacing was reduced.

**Figure 8:**
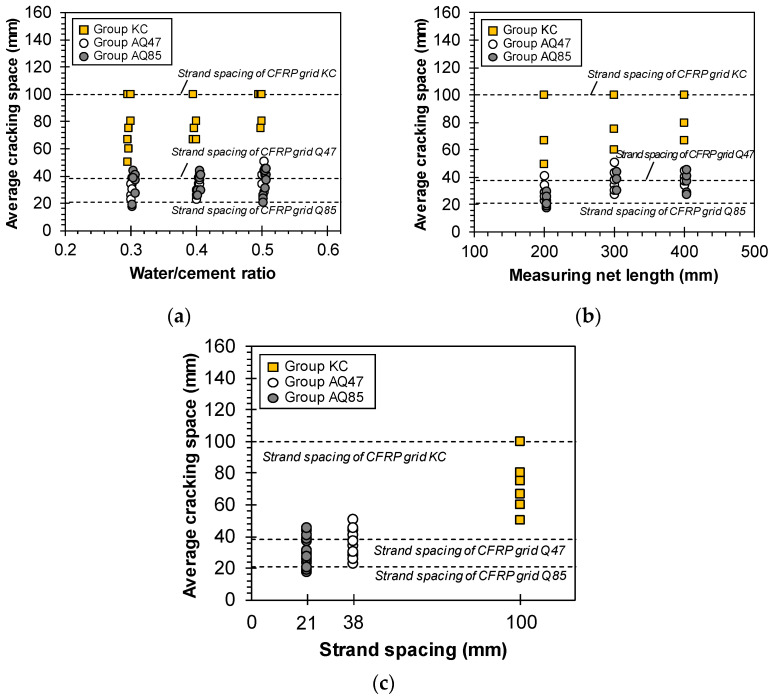
Average space of cracks with (**a**) w/c ratio, (**b**) net length, and (**c**) strand spacing of Groups KC, AQ47, and AQ85.

Figure 8 illustrates the crack characteristics of Groups KC, AQ47, and AQ85 based on TRM design variables such as mortar strength and specimen measuring net length as outlined in Figure 6. Figure 8a,b demonstrate that variations in mortar strength due to the w/c ratio and specimen measuring net length did not notably affect the average crack spacing. However, as depicted in Figure 8c, the average crack spacing tended to increase by up to 1.6 times as the strand spacing widened.

### 3.2. Tensile Stress–Strain Relationship

**Figure 9:**
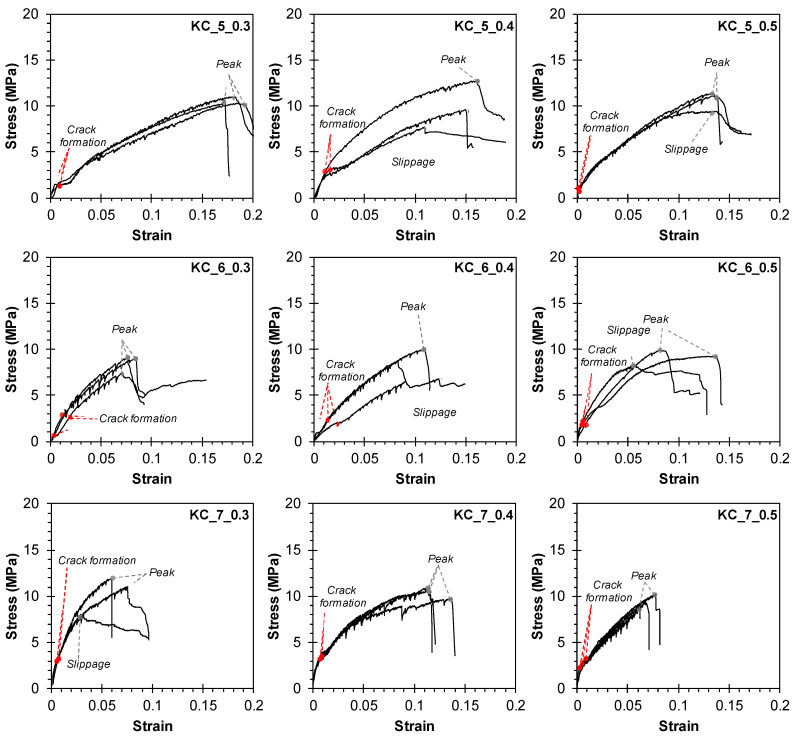
Relationship between tensile stress and strain of Group KC.

**Figure 10:**
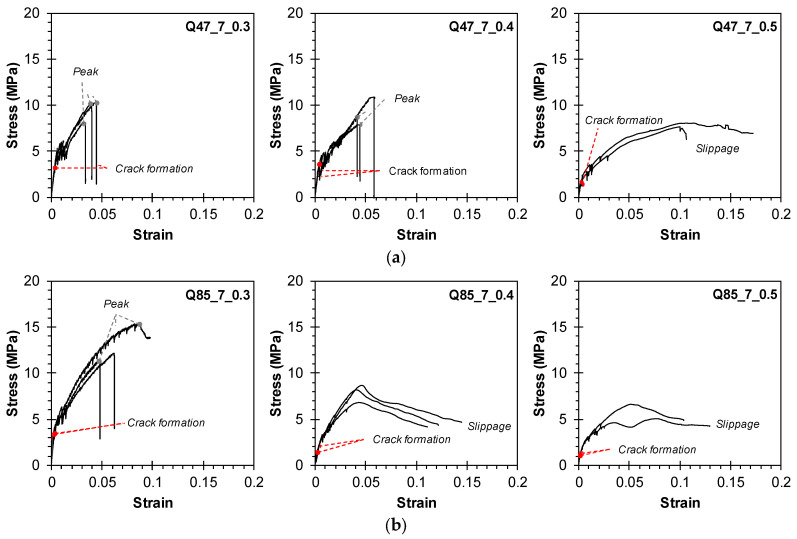
Relationship between tensile stress and strain: Groups (**a**) Q47 and (**b**) Q85.

**Figure 11:**
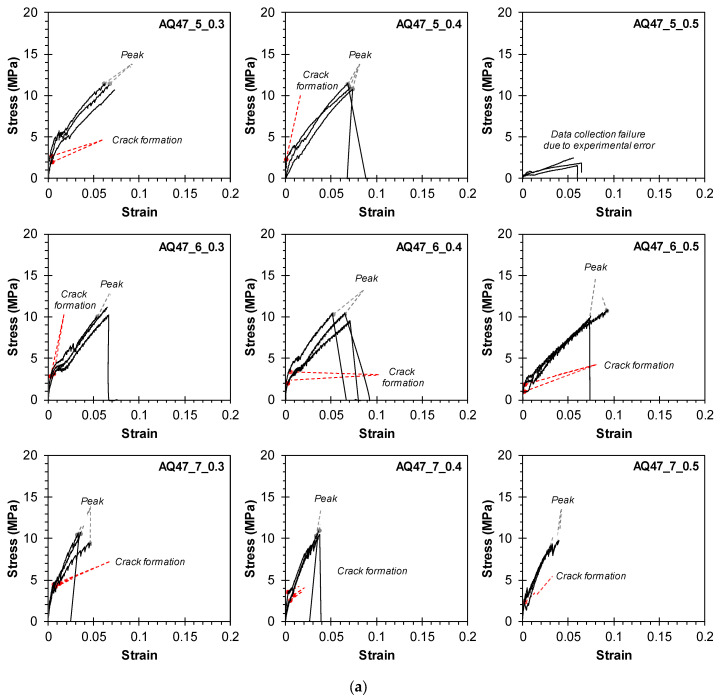

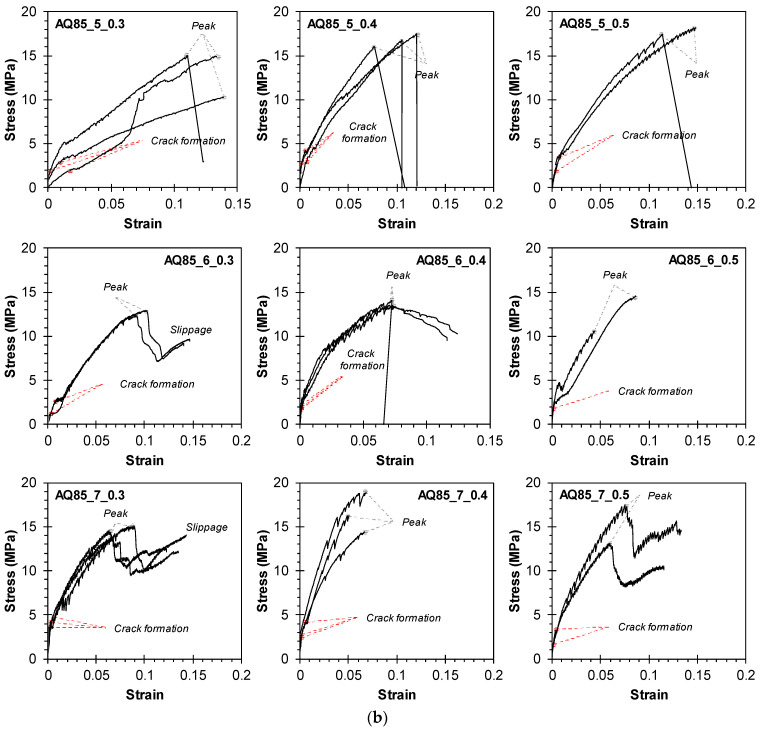
Relationship between tensile stress and strain: Groups (**a**) AQ47 and (**b**) AQ85.

Figure 9, Figure 10 and Figure 11 depict the tensile stress–strain relationships of triplicate or duplicate TRM specimens. Tensile stress was calculated by dividing the load value measured by the load cell by the cross-sectional area of the TRM specimens (140 mm × 20 mm for Group KC, 100 mm × 20 mm for Groups Q47, Q85, AQ47, and AQ85). Strain was determined by dividing the displacement measured by the WTDT by the net length of the specimen (200, 300, and 400 mm). The interaction between the mortar and the CFRP grid can have a decisive impact on the tensile behavior of TRM, alongside mechanical properties, potentially leading to a multifaceted composite stress–strain relationship [38,39].

Table 4 lists the cracking ultimate strengths of TRM specimens based on the stress–strain relationship. Cracking strength was identified as the point at which the specimen’s stiffness decreased, evidenced by a reduction in the slope of the stress–strain curve. The ultimate strength represents the maximum stress achieved before specimen failure or slippage. The coefficient of variation (COV) was calculated using measurements from the triplicate or duplicate TRM specimens.

For group KC, cracks typically occurred at 8.8–32.7% of the ultimate strength. Upon crack formation, the load transferred to the longitudinal strands in the tensile direction, resulting in reduced stiffness until reaching the ultimate strength, as illustrated in Figure 9. The load sharply dropped upon specimen fracture. However, specimens (KC_5_0.4, 0.5, KC_6_0.4, 0.5) experiencing slippage between the clamping steel plate and the TRM specimen (failure mode C in Figure 7c) exhibited a gradual reduction in load after reaching the ultimate strength.

Figure 10a,b depict the tensile behavior of Groups Q47 and Q85 using untreated biaxial-warp-knitting-structure CFRP grids. The cracking stress ranged from approximately 17.1% to 43.7% of the ultimate strength. When using the Q47 grid, which features a relatively wider strand spacing and greater contribution to concrete strength, the cracking strength was relatively high compared with the ultimate strength. Specimens Q47_7_0.3, 0.4, and Q85_7_0.3, benefiting from high mortar compressive strength due to a low w/c ratio, exhibited a sharp decrease in load upon rupture of the CFRP grid strands. In contrast, other specimens showed a gradual decrease in stress due to slippage in the clamping area. These stress results correlate with the observed final failure modes.

Figure 11a,b illustrate the tensile behavior of Groups AQ47 and AQ85 using sand-coated CFRP grids. For Specimen AQ47_5_0.5, the tensile stress–strain curve could not be obtained due to measurement errors. The application of sand coating on the surface of the CFRP grid significantly enhanced the bond quality between the CFRP grid and the mortar matrix by roughening the CFRP grid surface. This improvement led to substantially higher cracking strength and maximum stress. Specifically, the cracking stress for Groups AQ47 and AQ85 ranged from 16.2% to 44.5% and from 14.6% to 26.9% of the ultimate strength, respectively. Multiple cracks formed successively, causing the load to fluctuate as it transferred to the longitudinal strands in the tensile direction, thereby reducing stiffness. In Group AQ47, where the strand spacing of the CFRP grid was relatively wider compared with Group AQ85, higher stress was exerted on the strands due to fewer strands in the cross section. This resulted in a sharp decrease in load due to fractures at the central and end parts of the TRM specimen. Particularly in Group AQ85, some samples exhibited a decrease in load after partial fracture, followed by load support by the remaining longitudinal strands and subsequent slippage. Consequently, the stress–strain curve exhibited a resurgence, necessitating additional interpretation linked to the graph.

A comparison of the test results of Groups AQ47 and AQ85 with the tensile behaviors of Groups Q47 and Q85 shows that the sand coating on the surface of the CFRP grid effectively mitigates strand slippage in the clamping area. Based on the findings from Figure 10 and Figure 11 and Table 4, the tensile strength of TRM specimens appears to be primarily influenced by the interfacial bond strength between the CFRP grid and the mortar matrix. Therefore, the application of sand coating on the CFRP grid surface significantly enhances the interfacial bond performance between the mortar and the CFRP grid, underscoring its critical role in determining the tensile strength of TRM specimens.

**Figure 12:**
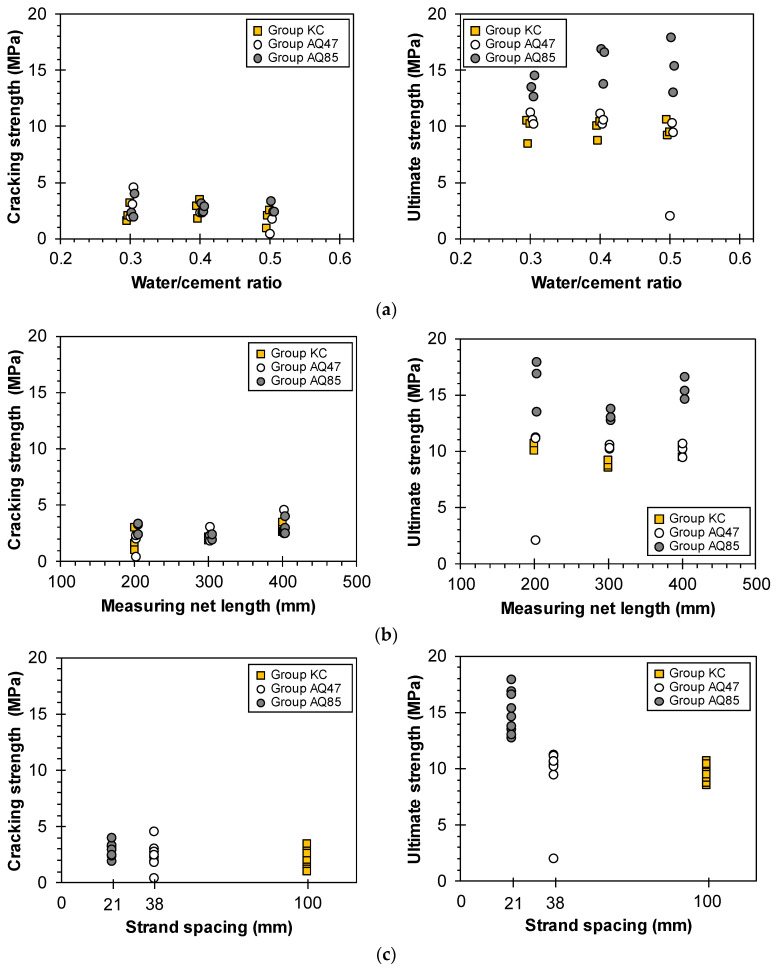
Cracking and ultimate strength of TRM with (**a**) w/c ratio, (**b**) net length of specimens, and (**c**) strand spacing.

Figure 12 demonstrates the cracking and ultimate strength of tensile specimens according to the design variables of the TRM specimens.

Figure 12a illustrates the strength characteristics based on the w/c ratio of the mortar matrix. Generally, as the w/c ratio increased, leading to reduced mortar compressive strength, the cracking strength decreased, while the ultimate strength remained largely unaffected by the mortar strength. Notably, Groups AQ85 and KC generally exhibited increases in their ultimate strengths despite the decrease in mortar strength, whereas Group AQ47 showed a tendency toward a decrease in ultimate strength with the decrease in mortar strength. Analyzing the strength characteristics relative to the area of individual strands remains consistent; Group AQ85, characterized by a higher density of strands due to narrower spacing, demonstrated an ultimate strength (tensile capacity) that exceeded that of Group AQ47 by 20.4% to 63.0%.

Analyzing the strength characteristics relative to the net length of the specimen, which correlates with the number of longitudinal strands, Figure 12b indicates that the cracking strength exhibited an increasing trend, but the ultimate strength exhibited a decreasing trend as the measuring net length increased in the case of Group AQ47, which exhibited failure modes A or B due to the ruptures of longitudinal strands. However, in the cases of Groups AQ85 and KC, which mainly exhibited failure modes C or D due to the slippage of the specimens, both the cracking and ultimate strengths did not exhibit a consistent trend similar to the crack patterns. This suggests that variations in specimen length did not notably contribute to crack control, and it is presumed that the ultimate strength may have been underestimated due to incomplete load transfer caused by slippage in the clamping area during the tensile testing process.

Figure 12c demonstrates that the spacing between the longitudinal strands had a more significant impact on the ultimate strength than the cracking strength. Group AQ85, with the narrowest strand spacing of 21 mm, exhibited the highest ultimate strength, ranging from 12.6 to 17.9 MPa, whereas Groups AQ47 and KC, with strand spacings of 38 mm and 100 mm, respectively, showed similar ultimate strengths ranging from 9.4 to 11.13 MPa. In the comparison of Groups AQ85 and AQ47, the strands had similar cross-sectional areas and tensile strengths. However, Group AQ85 had a larger number of strands, increasing the tensile capacity of CFRP grid AQ85 and resulting in a higher tensile strength than that of Group AQ47. Group KC, made from CFRP grid KC, exhibited the high tensile capacity with the largest cross-sectional area despite having the lowest tensile strength of the strands. Consequently, Group KC was predicted to have the highest tensile capacity. However, the final failures were attributed to slippage of the specimen or delamination of the mortar matrix within the load introduction areas (Table 3, Figure 7), with no strand ruptures observed. The strength of the mortar matrix was considered insufficient for CFRP grid KC, leading to a lower tensile strength in Group KC compared with Groups AQ85 and AQ47.

Therefore, a narrower spacing for longitudinal strands is advantageous for enhancing the ultimate strength.

**Figure 13:**
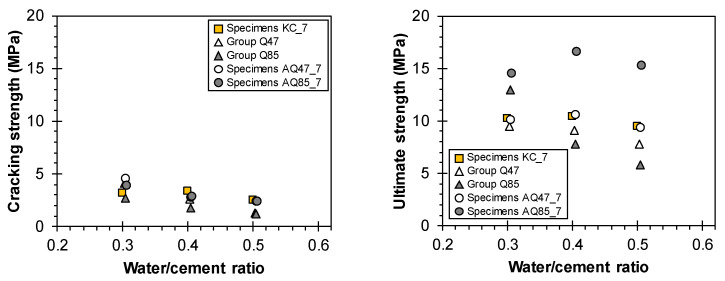
Cracking and ultimate strength of TRM with various carbon textile reinforcements.

**Figure 14:**
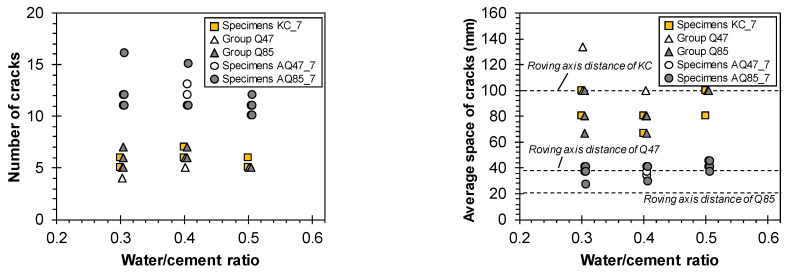
Crack properties of TRM with various carbon textile reinforcements.

Figure 13 and Figure 14 demonstrate the impact of CFRP grid characteristics on the cracking and ultimate strengths and crack properties for Specimen KC_7, Groups Q47 and Q85, and Specimens AQ47_7 and AQ85_7, all of which are 700 mm long. These results confirm that selecting and treating the CFRP grid appropriately can enhance tensile strength and improve crack resistance.

Generally, both the cracking and ultimate strengths decreased as the mortar compressive strength decreased, influenced by the w/c ratio (Figure 13). The variation in cracking strength due to changes in mortar strength was minimal, ranging from 1.36 to 4.49 MPa. In contrast, the ultimate strength exhibited significant differences depending on the characteristics of the CFRP grid, as it sustained load post-cracking until final failure occurred. Specimen AQ85_7, utilizing sand-coated CFRP grids, demonstrated the highest ultimate strength of 14.5 to 16.5 MPa. Interestingly, the CFRP grid KC used in TRM without sand coating achieved a similar ultimate strength to Specimen AQ47_7.

A sand coating on the surface of the CFRP grid significantly impacted the crack characteristics (Figure 14). Specimens AQ47_7 and AQ85_7, which utilized sand-coated CFRP grids, exhibited more than twice the number of cracks compared with other TRM specimens. The average spacing of cracks for Specimen AQ47_7 was predominantly within the 38 mm grid width of AQ47. By contrast, specimens with sand-uncoated CFRP grids showed cracks with spacings that exceeded the strand spacing. Increasing the number of cracks within the specimen’s measuring range reduces the average spacing of cracks and consequently decreases crack width [40,41], which is crucial for maintaining structural integrity.

### 3.3. Effect of CFRP Grid Characteristics on Bond Strengths

**Figure 15:**
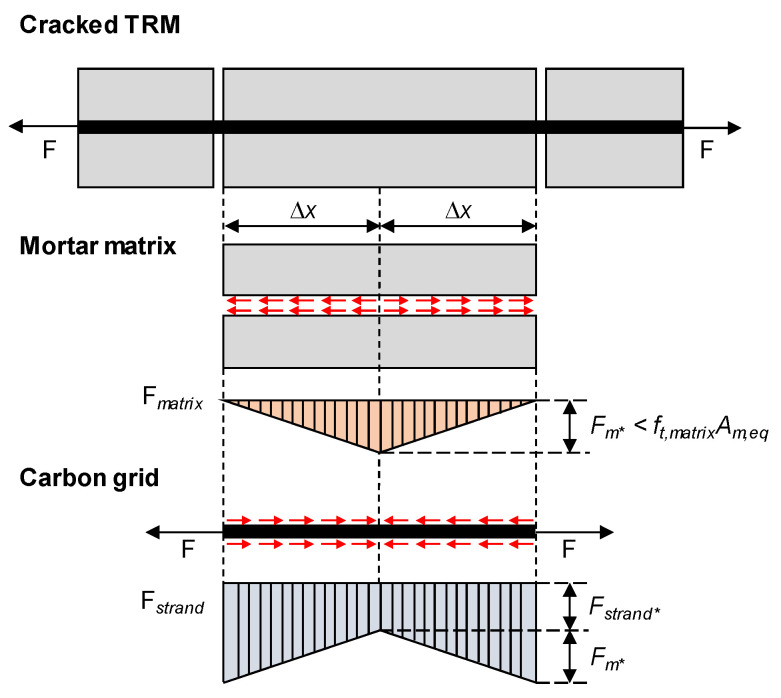
Stress diagram in cracked TRM.

The integration behavior between the mortar matrix and CFRP grid within the TRM specimens is crucial for the tensile performance of TRM. Therefore, estimating the average bond stress involves analyzing the crack pattern of TRM specimens. Theoretically, the applied load (F) along the specimen length is assumed to be shared between the mortar matrix (*F_matrix_*) and the stand of CFRP grid (*F_strand_*). When considering the segment of the specimen between two cracks, as the mortar matrix fails upon cracking, the entire load F is borne by the CFRP grid [37] (Figure 15). At the center of cracked TRM specimens, load transfer occurs through shear stress (red line) at the interface between the longitudinal strand and the mortar matrix, thereby reducing the contribution of the longitudinal strand. If shear stress is assumed to be constant across the specimen length, the load distribution between the mortar matrix and the longitudinal strand is represented by *F*_m*_ and *F*_strand*_. Assuming the maximum tensile stress of the mortar matrix equals the tensile strength of the mortar matrix, the average bond stress (*τ_m_*) can be evaluated using Equation (2):

(2) $$\tau_{m}=\frac{f_{t,\mathrm{matrix}}\cdot A_{m,\mathrm{eq}}}{∆x\cdot p_{\mathrm{strand}}\cdot n_{\mathrm{strand},\mathrm{long}}},$$

where *f_t_*_,*matrix*_ is the tensile strength of the mortar matrix, which was assumed to be 10% of the compressive strength [2], *A_m_*_,*eq*_ is the equivalent cross-sectional area of the mortar matrix, *∆x* is the distance between the cracks and the region reaching the tensile strength of the mortar matrix, *p_strand_* is the perimeter of the longitudinal strand within the cross section, and *n_strand_*_,*long*_ is the number of longitudinal strands in the cross section.

The perimeter length of the longitudinal strand in the cross section (p*_strand_*) is calculated as follows in Equation (3):

(3) $$p_{strand}=p_{\mathrm{Grid},\mathrm{strand}}\cdot n_{\mathrm{strand},\mathrm{long}}$$

where p*_Grid,strand_* is the perimeter of the strand and *n_strand_*_,*long*_ is the number of longitudinal strands in the cross section.

The equivalent cross-sectional area of the mortar (*A_m_*_,*eq*_) is calculated as follows in Equation (4).

(4) $$A_{m.\mathrm{eq}}=\frac{V_{\mathrm{TRM}}-V_{\mathrm{CT},\mathrm{grid}}}{l_{\mathrm{TRM}}},$$

where *V_TRM_* is the volume of the entire TRM specimen, *V_CT_*_,*grid*_ is the volume of the CFRP grid within the specimen, and *l_TRM_* is the length of the specimen. The volume of the CFRP grid within the specimen (*V_CT_*_,*grid*_) is calculated as follows in Equation (5):

(5) $$V_{\mathrm{CT},\mathrm{grid}}=A_{\mathrm{strand},\mathrm{long}}\cdot n_{\mathrm{strand},\mathrm{long}}\cdot l_{\mathrm{strand},\mathrm{long}}+A_{\mathrm{strand},\;\mathrm{trans}}\cdot n_{\mathrm{strand},\mathrm{long}}\cdot l_{\mathrm{strand},\mathrm{trans}}-w_{\mathrm{strand},\mathrm{long}}\cdot n_{\mathrm{strand},\mathrm{long}},$$

where *A_strand_*_, *long*_ and *A_strand_*_, *long*_ are the cross-sectional area and width of the longitudinal strand, *n_strand_*_,*long*_ and *l_strand_*_,*long*_ are the number and length of the longitudinal strands included in the cross section of the TRM specimen, respectively, *A_strand_*_, *trans*_ is the cross-sectional area of the transverse strand, and *n_strand_*_,*trans*_ and *l_strand_*_,*trans*_ are the number and length of the transverse strands included in the longitudinal section of the specimen, respectively.

*∆x* is calculated from Equation (6) based on the distance between two consecutive cracks measured from Equation (1) (*s_m_* in Table 3).

(6) $$∆x=\frac{s_{m}}{2}$$

**Figure 16:**
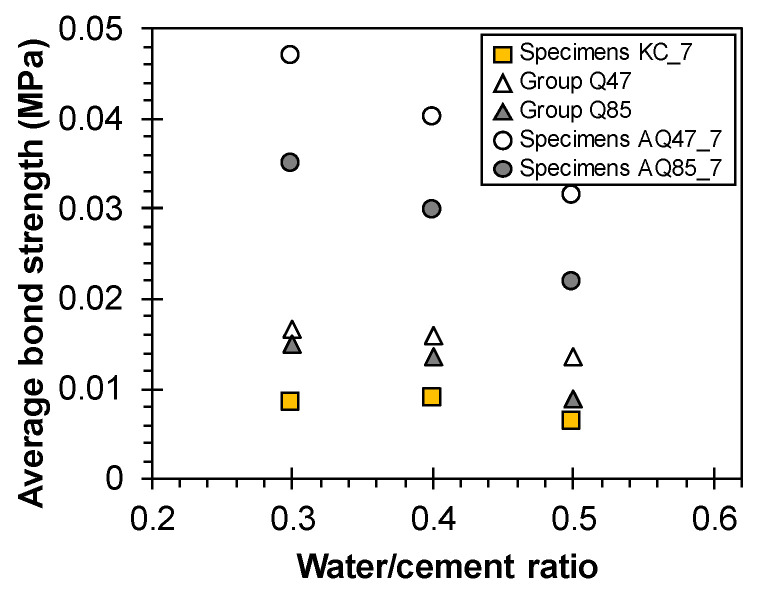
Average bond strength of TRM specimens.

The average bond stresses (*τ_m_*) of all the TRM specimens, predicted by substituting *∆x* calculated from Equation (6) into Equation (2), are listed in Table 5, along with detailed design parameters for each group. The effect of the TRM specimen design elements on *τ_m_* was analyzed for Specimen KC_7, Groups Q47 and Q85, and Specimens AQ47_7 and AQ85_7, each 700 mm long (see Figure 16).

Figure 16 clearly demonstrates that a sand coating on the surface of the CFRP grid improved bond strength by reducing the crack spacings. The *τ_m_* of sand-coated Specimens AQ47_7 and AQ85_7 was found to be 2.2–2.8 times higher than that of Specimens Q47 and Q85.

In particular, the effect of mortar tensile strength (*f_t_*_,*matrix*_) based on the w/c ratio of the mortar on *τ_m_* varied depending on the interface condition. Generally, *τ_m_* tended to decrease with decreasing *f_t_*_,*matrix*_. As the strength of the mortar matrix increased, enhanced interlocking with the sand coated on the surface of the CFRP grid also increased [42]. When internal interlocking occurs between the CFRP grid and the mortar matrix due to sand coating, the decrease in *τ_m_* with decreasing *f_t_*_,*matrix*_ becomes evident. However, for Specimens KC, Q47, and Q85, which have smooth surfaces, the change in *τ_m_* with decreasing *f_t_*_,*matrix*_ is negligible because they rely solely on inherent bond strength without interlocking elements on the grid surface.

In Group KC, the relatively large *∆x* calculated due to the more than 4.8-times higher *p_Grid_*_,*strand*_ and the 100 mm strand spacing compared with other CFRP grids is a factor that decreases bond strength. This calculation indicates that the dimensional characteristics of CFRP grid KC are disadvantageous for achieving strong bonding with the mortar matrix. However, the experimental results did not show the pullout or slippage of the CFRP grid due to reduced bond strength between the CFRP grid and the mortar matrix, and longitudinal strand rupture or mortar delamination in the load introduction area was observed. Therefore, future design considerations may require additional measures for the surface of CFRP grid KC to facilitate interlocking within the mortar matrix, alongside improvements in cross-sectional area to reduce *p_Grid_*_,*strand*_, and adjustments to strand spacing for better crack control.

## 4. Conclusions

This study investigated the impact of various design parameters on the tensile behavior of TRM specimens. These design elements include the type of carbon textile reinforcement, strength of the mortar matrix, measuring length of TRM specimens, and width of the carbon textile grid. The key findings are summarized as follows:

1. The direct tensile test results of TRM specimens revealed that, as cracks developed, the load shifted to the longitudinal strands of the CFRP grid, resulting in decreased stiffness until reaching the ultimate strength. Crack locations within the mortar matrix varied depending on specimen length and carbon textile type. Across most TRM specimens, once the ultimate strength was reached, a sharp decline was observed in load, coinciding with strand rupture. By contrast, specimens experiencing stress reduction from slippage between the clamping steel plate and TRM specimens showed a gradual load decrease after the ultimate strength. This was due to incomplete load transfer caused by clamping area slip, leading to an underestimation of the ultimate strength.
2. Increasing the number of longitudinal CFRP strands in TRM specimens’ cross sections effectively enhanced the ultimate strength up to 64.4% and crack control. Cracks uniformly appeared within the TRM specimen’s measurement range at intervals akin to the strands. As the strand spacing expanded, the average crack spacing also increased. When a sufficient number of strands are present within the TRM specimen, the consistency of the test is not significantly compromised, even with a shorter width-to-length ratio.
3. The strength of the mortar matrix significantly influenced the cracking strength of the TRM specimens more than their ultimate strength up to 27.4%. Changes in the transverse strands due to variations in the measuring net length of TRM specimens did not notably contribute to crack suppression but influenced the control over cracking space. Hence, effective evaluation of TRM specimen tensile performance appears achievable when the ratio of measuring net length to TRM width is relatively small (at least 2:1).
4. The surface coating of the CFRP grid is pivotal in determining the tensile strength and crack properties of TRM specimens. Sand coating notably enhanced the ultimate strength of TRM specimens by over 40% and effectively doubled the number of cracks compared with untreated specimens, leading to densely distributed cracks. Moreover, the sand coating on the CFRP grid surface improved interfacial bond performance between the CFRP grid and the mortar matrix by 2.2–2.8 times. This surface treatment introduced interlocking elements on the CFRP grid within TRM specimens, thereby enhancing interfacial bond performance as the mortar matrix strength increased.
5. TRM specimens incorporating CFRP grid KC with an adhesive structure did not exhibit optimal bond performance, thus failing to fully demonstrate its tensile potential. However, they showed relatively high tensile strength and similar failure modes compared with TRM specimens using other biaxial-warp-knitting-structure CFRP grids. Therefore, CFRP grid KC appears suitable for TRM applications. Future studies should focus on enhancing bond strength within the mortar matrix through coating applications, optimizing the grid width and cross-sectional shape, and exploring various other variables to further improve the tensile performance of TRM using CFRP grid KC.

## Figures and Tables

**Table 1:** Properties of CFRP grid.

CFRP Grid	Fiber Material	Structure	Impregnation Material	Surface Treatment	Cross-Sectional Area of Strand (mm^2^)	Strand Spacing (mm)	Tensile Strength (MPa)	Tensile Modulus of Elasticity (GPa)
KC	Carbon	Adhesive structure	Epoxy resin	-	20	100	2327	179
Q47	Carbon	Biaxial warp knitting structure	Epoxy resin	-	1.81	38	4379	255
AQ47								
Q85				Sand coating	1.81	21	4121	244
AQ85								

**Table 2:** Design parameters of TRM specimens.

Group	Specimen	Width (mm)	Thickness (mm)	Length (mm)	Measuring Net Length (mm)	w/c Ratio of Mortar	Number of Longitudinal Strands	Tensile Capacity (kN)
KC	KC_5_0.3	140	20	500	200	0.3	2	93.1
	KC_5_0.4					0.4		
	KC_5_0.5					0.5		
	KC_6_0.3	140	20	600	300	0.3		
	KC_6_0.4					0.4		
	KC_6_0.5					0.5		
	KC_7_0.3	140	20	700	400	0.3		
	KC_7_0.4					0.4		
	KC_7_0.5					0.5		
Q47	Q47_7_0.3	100	20	700	400	0.3	3	23.8
	Q47_7_0.4					0.4		
	Q47_7_0.5					0.5		
Q85	Q85_7_0.3	100	20	700	400	0.3	5	37.3
	Q85_7_0.4					0.4		
	Q85_7_0.5					0.5		
AQ47	AQ47_5_0.3	100	20	500	200	0.3	3	23.8
	AQ47_5_0.4					0.4		
	AQ47_5_0.5					0.5		
	AQ47_6_0.3	100	20	600	300	0.3		
	AQ47_6_0.4					0.4		
	AQ47_6_0.5					0.5		
	AQ47_7_0.3	100	20	700	400	0.3		
	AQ47_7_0.4					0.4		
	AQ47_7_0.5					0.5		
AQ85	AQ85_5_0.3	100	20	500	200	0.3	5	37.3
	AQ85_5_0.4					0.4		
	AQ85_5_0.5					0.5		
	AQ85_6_0.3	100	20	600	300	0.3		
	AQ85_6_0.4					0.4		
	AQ85_6_0.5					0.5		
	AQ85_7_0.3	100	20	700	400	0.3		
	AQ85_7_0.4					0.4		
	AQ85_7_0.5					0.5		

**Table 3:** Crack characteristics of TRM specimens.

Group	Specimen	Failure Mode (see Figure 7)	Number of Cracks, *n_crack_*			Average Space of Cracks, *s_m_* (mm)		
			*n_crack,1_*	*n_crack,2_*	*n_crack,3_*	*s_m,1_*	*s_m,2_*	*s_m,3_*
KC	KC_5_0.3	C, D	4	3	5	66.7	100.0	50.0
	KC_5_0.4	C, D	4	3	3	66.7	100.0	100.0
	KC_5_0.5	C, D	3	3	3	100.0	100.0	100.0
	KC_6_0.3	C, D	5	6	6	75.0	60.0	60.0
	KC_6_0.4	C, D	5	5	5	75.0	75.0	75.0
	KC_6_0.5	C, D	4	3	4	100.0	75.0	100.0
	KC_7_0.3	C, D	6	5	5	80.0	100.0	100.0
	KC_7_0.4	C, D	7	6	7	66.7	80.0	66.7
	KC_7_0.5	C, D	6	5	5	80.0	100.0	100.0
Q47	Q47_7_0.3	A, D	4	5	5	134.3	100.8	100.8
	Q47_7_0.4	A, B	5	5	6	100.8	100.8	80.6
	Q47_7_0.5	A	5	5		100.8	100.8	-
Q85	Q85_7_0.3	B	5	6	7	100.8	80.6	67.2
	Q85_7_0.4	C	7	6	7	67.2	80.6	67.2
	Q85_7_0.5	C	5	5		100.8	100.8	-
AQ47	AQ47_5_0.3	B	9	10	7	25.4	22.6	33.8
	AQ47_5_0.4	B	10	8	9	22.6	29.0	25.4
	AQ47_5_0.5	B	6	6	7	40.6	40.6	33.8
	AQ47_6_0.3	A, B	10	9	11	33.7	37.9	30.3
	AQ47_6_0.4	A, B	9	10	9	37.9	33.7	37.9
	AQ47_6_0.5	A, B	12	7	8	27.5	50.5	43.3
	AQ47_7_0.3	A, B	11	12	11	40.3	36.6	40.3
	AQ47_7_0.4	A, B	11	13	12	40.3	33.6	36.6
	AQ47_7_0.5	A, B	10	11	10	44.8	40.3	44.8
AQ85	AQ85_5_0.3	A, B	12	13	12	18.2	16.7	18.2
	AQ85_5_0.4	A, B	9	8	9	25.0	28.6	25.0
	AQ85_5_0.5	A, B	9	10	11	25.0	22.2	20.0
	AQ85_6_0.3	C	8	9	9	42.9	37.5	37.5
	AQ85_6_0.4	B, C	9	8	9	37.5	42.9	37.5
	AQ85_6_0.5	C, D	11	8	11	30.0	42.9	30.0
	AQ85_7_0.3	C, D	11	12	16	40.0	36.4	26.7
	AQ85_7_0.4	A	15	15	11	28.6	28.6	40.0
	AQ85_7_0.5	C, D	11	12	10	40.0	36.4	44.4

**Table 4:** Test results of TRM specimens.

Group	Specimen	Cracking Strength		Ultimate Strength		*f*_cr_/*f*_u_
		*f*_cr_ (MPa)	COV	*f*_u_ (MPa)	COV	
KC	KC_5_0.3	1.55	0.08	10.53	0.04	0.15
	KC_5_0.4	2.91	0.25	10.03	0.25	0.29
	KC_5_0.5	0.94	0.05	10.65	0.10	0.09
	KC_6_0.3	2.07	0.63	8.47	0.11	0.24
	KC_6_0.4	1.78	0.45	8.69	0.14	0.21
	KC_6_0.5	2.00	0.09	9.16	0.09	0.22
	KC_7_0.3	3.21	0.06	10.25	0.21	0.31
	KC_7_0.4	3.41	0.09	10.42	0.05	0.33
	KC_7_0.5	2.55	0.18	9.48	0.08	0.27
Q47	Q47_7_0.3	4.15	0.28	9.51	0.13	0.44
	Q47_7_0.4	2.69	0.21	9.16	0.17	0.29
	Q47_7_0.5 ^a^	1.35	0.06	7.87	0.04	0.17
Q85	Q85_7_0.3	2.76	0.06	13.01	0.16	0.21
	Q85_7_0.4	1.77	0.20	7.87	0.12	0.23
	Q85_7_0.5 ^a^	1.23	0.18	5.89	0.18	0.21
AQ47	AQ47_5_0.3	1.86	0.09	11.13	0.04	0.17
	AQ47_5_0.4	2.20	0.11	11.06	0.03	0.20
	AQ47_5_0.5	0.32	0.32	1.96	0.24	0.16
	AQ47_6_0.3	2.98	0.14	10.48	0.06	0.28
	AQ47_6_0.4	2.20	0.10	10.11	0.05	0.22
	AQ47_6_0.5	1.68	0.36	10.22	0.06	0.16
	AQ47_7_0.3	4.49	0.05	10.11	0.06	0.44
	AQ47_7_0.4	2.70	0.16	10.55	0.03	0.26
	AQ47_7_0.5	2.35	0.04	9.37	0.07	0.25
AQ85	AQ85_5_0.3	2.26	0.17	13.42	0.20	0.17
	AQ85_5_0.4	3.10	0.27	16.77	0.04	0.18
	AQ85_5_0.5 ^a^	3.25	0.35	17.85	0.03	0.18
	AQ85_6_0.3 ^a^	1.84	0.58	12.62	0.03	0.15
	AQ85_6_0.4	2.32	0.09	13.70	0.02	0.17
	AQ85_6_0.5	2.30	0.20	12.95	0.26	0.18
	AQ85_7_0.3	3.89	0.12	14.48	0.04	0.27
	AQ85_7_0.4	2.81	0.22	16.53	0.14	0.17
	AQ85_7_0.5 ^a^	2.36	0.14	15.27	0.20	0.15

^a^ Test results were derived from two specimens.

**Table 5:** Detailed parameters for evaluation of average bond strength.

Group	Specimen	*p_Grid_*_,*strand*_ (mm)	*n_strand_*_,*long*_	*A_m,eq_* (mm^2^)	*∆x* (mm)			*f_t,matrix_* (MPa)	Average Bond Strength	
					1	2	3		*τ_m_* (MPa)	COV
KC	KC_5_0.3	42	2	2690	33.3	50.0	25.0	4.9	0.023	0.33
	KC_5_0.4	42	2	2690	33.3	50.0	50.0	3.9	0.015	0.25
	KC_5_0.5	42	2	2690	50.0	50.0	50.0	3.6	0.012	0.00
	KC_6_0.3	42	2	2704	37.5	30.0	30.0	4.9	0.016	0.12
	KC_6_0.4	42	2	2704	37.5	37.5	37.5	3.9	0.011	0.00
	KC_6_0.5	42	2	2704	50.0	75.0	50.0	3.6	0.007	0.22
	KC_7_0.3	42	2	2711	40.0	50.0	50.0	4.9	0.008	0.13
	KC_7_0.4	42	2	2711	33.3	40.0	33.3	3.9	0.009	0.10
	KC_7_0.5	42	2	2711	40.0	50.0	50.0	3.6	0.006	0.13
Q47	Q47_7_0.3	8.8	3	1986	66.7	50.0	50.0	4.9	0.017	0.16
	Q47_7_0.4	8.8	3	1986	50.0	50.0	40.0	3.9	0.016	0.13
	Q47_7_0.5 ^a^	8.8	3	1986	50.0	50.0	0.0	3.6	0.014	0.00
Q85	Q85_7_0.3	8	5	1976	50.0	40.0	33.3	4.9	0.015	0.20
	Q85_7_0.4	8	5	1976	33.3	40.0	33.3	3.9	0.014	0.10
	Q85_7_0.5 ^a^	8	5	1976	50.0	50.0	0.0	3.6	0.009	0.00
AQ47	AQ47_5_0.3	8.8	3	1983	12.7	11.3	16.9	4.9	0.138	0.20
	AQ47_5_0.4	8.8	3	1983	11.3	14.5	12.7	3.9	0.116	0.12
	AQ47_5_0.5	8.8	3	1983	20.3	20.3	16.9	3.6	0.072	0.11
	AQ47_6_0.3	8.8	3	1986	16.8	18.9	15.1	4.9	0.072	0.11
	AQ47_6_0.4	8.8	3	1986	18.9	16.8	18.9	3.9	0.054	0.07
	AQ47_6_0.5	8.8	3	1986	13.8	25.2	21.6	3.6	0.048	0.33
	AQ47_7_0.3	8.8	3	1986	20.1	18.3	20.1	4.9	0.047	0.06
	AQ47_7_0.4	8.8	3	1986	20.1	16.8	18.3	3.9	0.040	0.09
	AQ47_7_0.5	8.8	3	1986	22.4	20.1	22.4	3.6	0.032	0.06
AQ85	AQ85_5_0.3	8	5	1970	9.3	8.5	9.3	4.9	0.132	0.05
	AQ85_5_0.4	8	5	1970	12.8	14.6	12.8	3.9	0.072	0.08
	AQ85_5_0.5 ^a^	8	5	1970	12.8	11.4	10.3	3.6	0.078	0.11
	AQ85_6_0.3 ^a^	8	5	1974	21.8	19.1	19.1	4.9	0.040	0.08
	AQ85_6_0.4	8	5	1974	19.1	21.8	19.1	3.9	0.032	0.08
	AQ85_6_0.5	8	5	1974	15.3	21.8	15.3	3.6	0.035	0.19
	AQ85_7_0.3	8	5	1976	20.3	18.4	13.5	4.9	0.036	0.22
	AQ85_7_0.4	8	5	1976	14.5	14.5	20.3	3.9	0.030	0.18
	AQ85_7_0.5 ^a^	8	5	1976	20.3	18.4	22.5	3.6	0.022	0.10

^a^ Test results were derived from two specimens.

## Data Availability

The original contributions presented in the study are included in the article, further inquiries can be directed to the corresponding author.
